# Editorial: Mesh Complications in Hernia Surgery

**DOI:** 10.3389/fsurg.2022.841672

**Published:** 2022-03-07

**Authors:** Friedrich Kallinowski, René H. Fortelny, Ferdinand Köckerling, Franz Mayer, Salvador Morales-Conde, Gabriel Sandblom

**Affiliations:** ^1^General, Visceral and Transplantation Surgery, University Hospital, Heidelberg, Germany; ^2^General Surgery/Medical Faculty, Sigmund Freud Private University, Vienna, Austria; ^3^Hernia Center, Vivantes Humboldt-Hospital, Charité University Medicine, Berlin, Germany; ^4^Department of General, Visceral and Thoracic Surgery, University Hospital Salzburg, Paracelsus Medical University, Salzburg, Austria; ^5^Department of Surgery, General Hospital Hallein, Hallein, Austria; ^6^Unit of Innovation in Minimally Invasive Surgery, University Hospital Virgen del Rocio, University of Sevilla, Sevilla, Spain; ^7^Unit of General and Digestive Surgery, Hospital Quironsalud Sagrado Corazon, Sevilla, Spain; ^8^Department of Clinical Science and Education Södersjukhuset, Karolinska Institutet, Stockholm, Sweden; ^9^Department of Surgery, Södersjukhuset, Stockholm, Sweden

**Keywords:** hernia repair, GRIP concept, cyclic loading hernia, mesh materials, bench test hernia, incisional abdominal ventral hernia, CT abdomen with Valsalva, unstable abdominal wall

Hernia repair aims at restoring the integrity of the abdominal wall and its load-bearing capacity. These aims are sought after with textile meshes augmenting the herniated abdominal wall. The results are plagued by seroma formation, infections, pain and recurrences. Surgeons, patients, hospital systems, and health policy makers throughout the world are eagerly seeking better solutions.

Basic science in hernia research points toward genetic changes generating weak collagen (1). Weak collagens are unable to sufficiently bear load (2). An unstable abdominal wall can result. Advanced suture techniques can prevent most but not all burst abdomen and hernia (3). During the wound healing, skin and subcutaneous tissue will cover the weak fascia-forming collagen. The fascial dehiscence will be invisible from the outside. The repaired abdominal wall is thus open to hernia formation (4). In due course, the dehiscence creates an instability with the biological consequence of a seroma formation (5). A seroma easily gets infected with the wound infection further facilitating hernia recurrence. The aggressiveness of bacteria is an important influence on the infectious load (6). Pain is caused by small nerve fibers within the wound being inflicted by microbial products such as lactic acid and by the stretching of the lax collagen beyond a fraction of a millimeter. A recurrence is the obvious end of a mechanical overload.

We cannot change our genetic fittings or the aggressiveness of the microbiome surrounding us. But we can create stronger defect closures. Material science advises us that pulse loads act as the destructive force for compounds made from polymers such as tissues and textiles. In this sense, strong defect closures can be defined as those which withstand pulse loads. In mankind, pulse loads are caused by coughing, jumping, sharply bending and other motions. In order to get a strong defect closure, a load limit needed to be defined (7). An overload by daily activities should be prevented as long as the incised abdominal wall heals. For this sake, an analysis of daily activities and the benefits of abdominal binders are desperately needed (8).

Not all people are equal. Tissue quality needs to be assessed in the individual patient prior to hernia repair. Frail people and athletes might be two cornerstones marking the continuum of ordinary people, workers or hernia patients. The analysis of the individual tissue quality has become possible with the use of a bench test for cyclic loading and advanced imaging techniques (9). The concept of an unstable abdominal wall repaired by an individualized biomechanical approach was condensed in the GRIP concept [gained resistance toward impact related to pressure; (10)]. This approach is based on the milestone consideration that not the mesh or an overlap *per se*, but the mesh-defect-area ratio (MDAR) provide the fundamental base for a durable repair (11).

Not all meshes or fixation elements are equal (12). Advanced mesh material with well-known biomechanical properties combined with sophisticated techniques can give excellent clinical results (9, 13, 14). Material science works with coefficients permitting engineers to build skyscrapers, supersonic airplanes or just fitting a balcony to a house in an earthquake area. Surgeons need such coefficients for the materials they use. At this point in time, the first data are available. In the future, most materials will be tested since regulatory agencies, patients and health policy makers seek more durable repairs. Surgeons can fulfill this wish once the required data are available (14).

Mesh-related complications are costly and can appear years after hernia repair (15). Long-term follow up requires registries such as Herniamed^®^ (16, 17). Such a registry can be extended for research purposes following patients for years (14). Patient-reported outcomes should be included (18). Patient's wishes and expectations matter and should be considered for good outcomes (19).

The hernia size has to be considered in abdominal wall reconstructions (20). Mesh material properties are critically underreported, depriving surgeons and patients alike from the benefits of optimal surgical techniques (21). Nobody would repair an airplane wing with sticky tape, some glue off the shelf and prayers. In contrast, well-tested materials are used in aviation industries by highly trained professionals according to standard procedures. Such a strategy will be a future road to avoid complications falsely attributed to the mesh. Preventing instability of both the abdominal wall and the mesh repair is an obvious path to more durable reconstructions in incisional hernia. The findings detailed above may be generalized to other types of hernia after future research. Instead of blaming the meshes, the reconstructions as compound structures must safely bear load during the healing process. With a Research Topic on “Mesh-related complications,” Frontiers in Surgery contributed to an ongoing discussion how make hernia repair better in the future.

## Author Contributions

All authors listed have made a substantial, direct, and intellectual contribution to the work and approved it for publication.

## Conflict of Interest

The authors declare that the research was conducted in the absence of any commercial or financial relationships that could be construed as a potential conflict of interest.

## Publisher's Note

All claims expressed in this article are solely those of the authors and do not necessarily represent those of their affiliated organizations, or those of the publisher, the editors and the reviewers. Any product that may be evaluated in this article, or claim that may be made by its manufacturer, is not guaranteed or endorsed by the publisher.

## References

[1] Franz MG. The biology of hernias and the abdominal wall. Hernia. (2006) 10:462–71. 10.1007/s10029-006-0144-9 17006625

[2] Münster S Jawerth LM Leslie BA Weitz JI Fabry B Weitz DA. Strain history dependence of the nonlinear stress response of fibrin and collagen networks. Proc Natl Acad Sci U S A. (2013) 110:12197–202. 10.1073/pnas.1222787110 23754380 PMC3725119

[3] Albertsmeier M Hofmann A Baumann P Riedl S Reisensohn C Kewer JL. Effects of the short-stitch technique for midline abdominal closure: short-term results from the randomised-controlled ESTOIH trial. Hernia. (2021). 10.1007/s10029-021-02410-y [Epub ahead of print]. PMC8881264 34050419

[4] Pollock AV Evans M. Early prediction of late incisional hernias. Br J Surg. (1989) 76:953–4. 10.1002/bjs.1800760926 2804595

[5] Morales-Conde S Gómez-Menchero J Alarcón I Balla A. Retroprosthetic seroma after laparoscopic ventral hernia repair is related to mesh used? J Laparoendosc Adv Surg Tech A. (2020) 30:241–5. 10.1089/lap.2019.0646 31742465

[6] Xu X Zhan M Li X Chen T Yang L. In vivo analysis of the resistance of the meshes to escherichia coli infection. Front Surg. (2021) 8:644227. 10.3389/fsurg.2021.644227 34250004 PMC8264128

[7] Kallinowski F Ludwig Y Löffler T Vollmer M Lösel PD Voß S. Biomechanics applied to incisional hernia repair – Considering the critical and the gained resistance towards impacts related to pressure. Clin Biomech (Bristol, Avon). (2021) 82:105253. 10.1016/j.clinbiomech.2020.105253 33401197

[8] Schaaf S Schwab R Güsgen C Vilz TO Willms A. Recommendations on postoperative activities after abdominal operations and incisional hernia repair-a national and international survey. Front Surg. (2021) 8:713138. 10.3389/fsurg.2021.713138 34660675 PMC8511488

[9] Kallinowski F Ludwig Y Gutjahr D Gerhard C Schulte-Hörmann H Krimmel L. Biomechanical influences on mesh-related complications in incisional hernia repair. Front Surg. (2021) 8:763957. 10.3389/fsurg.2021.763957 34778367 PMC8586217

[10] Kallinowski F Gutjahr D Harder F Sabagh M Ludwig Y Lozanovski VJ. The grip concept of incisional hernia repair-dynamic bench test, ct abdomen with valsalva and 1-year clinical results. Front Surg. (2021) 8:602181. 10.3389/fsurg.2021.602181 33937312 PMC8080034

[11] Tulloh B de Beaux A. Defects and donuts: the importance of the mesh: defect area ratio. Hernia. (2016) 20:893–5. 10.1007/s10029-016-1524-4 27585805

[12] Olsson A Kiwanuka O Sandblom G Stackelberg O. Evaluation of functional outcomes following rectus diastasis repair-an up-to-date literature review. Hernia. (2021) 25:905–14. 10.1007/s10029-021-02462-0 34302558 PMC8370918

[13] 13) Zhang W Zhao Y Shao X Cheng T Ji Z Li J. Long-term follow-up of lichtenstein repair of inguinal hernia in the morbid patients with self-gripping mesh (ProgripTM). Front Surg. (2021) 8:748880. 10.3389/fsurg.2021.748880 34722625 PMC8554065

[14] Nessel R Löffler T Rinn J Lösel P Voss S Heuveline V. Primary and recurrent repair of incisional hernia based on biomechanical considerations to avoid mesh-related complications. Front Surg. (2021) 8:764470. 10.3389/fsurg.2021.764470 34977141 PMC8714753

[15] Zhang J Hu Z Lin X Chen B. Late-onset ileocutaneous fistula eight years after plug repair with polypropylene mesh: a case report. Front Surg. (2021) 8:785087. 10.3389/fsurg.2021.785087 34869573 PMC8634260

[16] Köckerling F Sheen AJ Berrevoet F Campanelli G Cuccurullo D Fortelny R. The reality of general surgery training and increased complexity of abdominal wall hernia surgery. Hernia. (2019) 23:1081–91. 10.1007/s10029-019-02062-z 31754953 PMC6938469

[17] KKöckerling F Brunner W Fortelny R Mayer F Adolf D Niebuhr H. Treatment of small (<2 cm) umbilical hernias: guidelines and current trends from the Herniamed Registry. Hernia. (2021) 25:605–17. 10.1007/s10029-020-02345-w 33237505

[18] East B Hill S Dames N Blackwell S Laidlaw L Gök H. patient views around their hernia surgery: a worldwide online survey promoted through social media. Front Surg. (2021) 8:769938. 10.3389/fsurg.2021.769938 35004837 PMC8739190

[19] Fadaee N Huynh D Towfigh S. #Mesh: Social media and its influence on perceptions in hernia repair. Am Surg. (2020) 86:1351–7. 10.1177/0003134820964459 33103471

[20] Parker SG Halligan S Erotocritou M Wood CPJ Boulton RW Plumb AAO. A systematic methodological review of non-randomised interventional studies of elective ventral hernia repair: clear definitions and a standardised minimum dataset are needed. Hernia. (2019) 23:859–72. 10.1007/s10029-019-01979-9 31152271 PMC6838456

[21] Kahan LG Blatnik JA. Critical under-reporting of hernia mesh properties and development of a novel package label. J Am Coll Surg. (2018) 226:117–25. 10.1016/j.jamcollsurg.2017.10.020 29133265

